# Multidrug-resistant bacteria in a paediatric palliative care inpatient unit: results of a one year surveillance

**DOI:** 10.3205/dgkh000338

**Published:** 2020-02-19

**Authors:** Pia Schmidt, Carola Hasan, Arne Simon, Christine Geffers, Julia Wager, Boris Zernikow

**Affiliations:** 1 Witten/Herdecke University, Faculty of Health, School of Medicine, Department of Children’s Pain Therapy and Paediatric Palliative Care, Datteln, Germany; 2 Paediatric Palliative Care Centre, Children’s and Adolescents’ Hospital Datteln, Datteln, Germany; 3 Saarland University Medical Center and Saarland University Faculty of Medicine, Department of Paediatric Haematology and Oncology, Homburg/Saar, Germany; 4 Institute of Hygiene and Environmental Medicine, Charité – University Medicine in Berlin, Campus Benjamin Franklin, Berlin, Germany

**Keywords:** paediatric palliative care, MDR pathogen, nosocomial infection, surveillance

## Abstract

**Aim:** Nosocomial infections (NIs) and multidrug resistant (MDR) pathogens are an important paediatric healthcare issue. In vulnerable patients such as children with life-limiting conditions, MDR infections can be life-threatening. Additionally, these children have a significantly increased risk for colonisation with MDR pathogens. Therefore, it is vital to prevent new colonisations with MDR pathogens in this vulnerable patient group. However, little is known about colonisation with MDR pathogens and NIs in inpatient units for paediatric palliative care (PPC). The aim of this study was to investigate the prevalence of colonisation with MDR pathogens and the incidence of NIs in a PPC unit.

**Methods:** Evaluation of surveillance data of a PPC unit. All patients admitted to a PPC unit from 1^st^ April 2012 to 31^st^ March 2013 were screened for MDR pathogens upon admission. Patients who exhibited clinical signs of an infection during their inpatient stay were screened again.

**Results:** During the study period, 198 cases were admitted to the unit. Those cases represent 118 patients. 18% of the patients were colonised with MDR pathogens. The most common MDR pathogens were *E. coli* (8.1%) and *Pseudomonas ssp.* (8.1%). In addition, 58% of patients with tracheostomy had MDR pathogens in their tracheal secretions. The incidence density of NIs was 0.99 per 1000 inpatient treatment days with no NI caused by MDR pathogens.

**Conclusion:** Due to a high prevalence, it is reasonable to screen PPC patients for MDR pathogen colonisation before or during admission. Special attention must be given to patients with tracheostomy. Our results provide preliminary evidence that participation in social activities in a PPC unit for patients colonised with MDR pathogens is safe if hygiene concepts are applied.

## Zusammenfassung

**Ziel:** Nosokomiale Infektionen (NI) und multiresistente Erreger (MRE) stellen große Herausforderungen der modernen pflegerischen und medizinischen Versorgung dar. Besonders für Kinder und Jugendliche mit lebenslimitierenden Erkrankungen kann eine MRE bedingte Infektion lebensbedrohlich sein. Zudem haben diese Kinder ein erhöhtes Risiko mit einem MRE besiedelt zu sein. Daher ist es für die betroffenen Kinder und Jugendlichen notwendig, neue Kolonisationen mit einem MRE zu vermeiden. Da bisher wenig bekannt ist über die Besiedlung der Patienten einer Kinderpalliativstation mit MRE ebenso wie über die Anzahl von NI während eines stationären Aufenthaltes, ist es das Ziel der Studie, die Prävalenz von MRE besiedelten Patienten sowie die Anzahl der NI auf einer Kinderpalliativstation zu erheben.

**Methode:** Die Daten wurden im Rahmen einer Surveillance erhoben. Alle Patienten, die vom 01.04.2012 bis 31.03.2013 stationär auf eine Kinderpalliativstation aufgenommen wurden, wurden am Tag der Aufnahme auf MRE gescreent. Entwickelte ein Patient klinische Anzeichen einer Infektion wurden erneut Abstriche auf MRE entnommen.

**Ergebnisse:** Insgesamt gab es im Studienzeitraum 198 Aufnahmen von 118 unterschiedlichen Patienten auf die Kinderpalliativstation. 18% der Patienten waren mit einem MRE besiedelt. Am häufigsten traten* E. coli* (8.1%) und *Pseudomonas ssp.* (8.1%) auf. Bei 58% der Patienten mit einem Tracheostoma konnte ein MRE im Trachealsekret nachgewiesen werden. Die Inzidenzdichte der NI lag bei 0,99 pro 1000 Patiententage. Es trat keine NI durch einen MRE auf.

**Schlussfolgerung:** Die hohe Anzahl an MRE besiedelten Patienten auf der Kinderpalliativstation spricht dafür, pädiatrische Palliativpatienten vor einem stationären Aufenthalt auf MRE zu screenen. Besonders sind dabei die Patienten mit einem Tracheostoma zu beachten. Unsere Ergebnisse geben erste Hinweise darauf, dass soziale Teilhabe auf der pädiatrischen Palliativstation für Patienten mit MRE Besiedlung sicher ist, wenn entsprechende Hygienekonzepte angewandt werden.

## Introduction

Nosocomial infections (NIs) are a major complication of (paediatric) hospital care [1], [2]. They increase morbidity and mortality and are associated with prolonged hospital stay [3]. 

Paediatric palliative care (PPC) focuses on children and adolescents with life-limiting conditions (LLC). Most of the patients suffer from non-oncological diseases [4]. They require PPC intermittently for several years and not just at the end of life [5]. These patients are at risk for colonisation with multidrug-resistant (MDR) pathogens due to previous hospital stays, surgical interventions, frequent antibiotic treatments, presence of long-lasting devices (e.g., percutaneous endoscopic gastrostomy [PEG] tube, central venous catheter, tracheostomy) [6], and their complex main condition. Due to high utilisation rates of long-lasting devices and antimicrobial and immunosuppressive therapies, PPC patients face an increased risk of experiencing NIs [7], [8]. For many children with LLC, any acute infection of the lower respiratory tract can be life threatening [9]. Empirical antibiotic treatment of NIs may be complicated in this particular population due to an increased incidence of NI caused by MDR pathogens. In this regard, nosocomial transmission of pathogens – in particular – MDR must be avoided in PPC units. 

Despite the high clinical importance of MDR pathogens in the PPC setting, little is known about its prevalence and the incidence of NI. Therefore, this study investigates the prevalence of colonisation with MDR pathogens in patients admitted to a German PPC inpatient unit as well as the number of NIs during the inpatient stay.

## Methods

This surveillance was carried out in the PPC unit at the Children’s and Adolescents’ Hospital Datteln, Witten/Herdecke University, Germany, during a 12-month period from 1^st^ April 2012 to 31^st^ March 2013.

### Patients and setting

The PPC unit at the Children’s and Adolescents’ Hospital Datteln, Witten/Herdecke University, Germany, is not a children’s hospice but a separate, self-reliant palliative care unit in a tertiary care university children’s hospital. The PPC unit offers intensive paediatric palliative hospital care but not respite care. 

The hygiene care concept for patients who are colonised with MDR pathogens implies that all patients of the PPC unit are screened for colonisation with MDR pathogens by their general practitioner/outpatient paediatrician prior to admission. If screening results are lacking on the day of admission, patients will be screened by the staff of the PPC unit. Strict barrier nursing is performed until screening results are available (on average 48–72 h). If screening results confirm a colonisation with MDR pathogens, patients must remain under barrier nursing during the whole inpatient stay. If screening results do not confirm a colonisation with MDR pathogens, barrier nursing will be lifted. Standard precautions are applied to each patient contact; hand disinfection is performed with Sterilium (Bode Inc. Heidenheim) [10]. Hence, patients who are colonised with MDR pathogens are allowed to participate in social activities, e.g., music or art therapy, in the unit while wearing gowns, following the regulations of barrier nursing and applying strict hand disinfection. The attending nurse supervises compliance with these targeted precautions. The complete hygiene concept has been published elsewhere [11]. 

### Surveillance 

This study included all admissions to the PPC unit during a 12-month period from 1^st^ April 2012 to 31^st^ March 2013 (n=198). During the study period, the prospective surveillance of NIs was performed following standard definitions [12]. In addition, all patients of the PPC unit were screened by their general practitioner/outpatient paediatrician immediately prior to admission or upon admission to detect any colonisation with MDR pathogens. Upon admission to the PPC unit, a smear utensil set (Nerbe Inc. Winsen/Luhe, Germany) with a viscose-flocked plastic stick and Amies Agar Gel Medium without charcoal was used to transport swabs [13]. Respiratory secretions (including tracheal secretions in patients with tracheostomy) were sampled with a tracheal suction kit (Dahlhausen Inc., Cologne, Germany) [14]. The MDR pathogen screening samples were obtained from both nostrils (Methicillin-resistant Staphylococcus aureus [MRSA]), throat (MRSA, multidrug-resistant Gram-negative pathogens [MRGN]), anal/perianal region (MRGN) and, if applicable, wounds and entrance site of devices, e.g., PEG or central venous catheter (MRSA and MRGN). In the case of chronic lung conditions, a sputum sample was analysed. In patients who exhibited clinical signs of an infection during their inpatient stay, samples were taken again from the same sites, and a blood culture was performed if an infection was clinically indicated. 

MRGN are defined as follows: Gram-negative pathogens with in vitro resistance to at least 2 of 4 antibiotic groups (Extended spectrum penicillin [piperacillin], third/fourth generation cephalosporins, fluoroquinolones, carbapenems) were allocated to the category “MRGN” regardless of the mechanism of resistance (e.g., extended-spectrum beta-lactamase production) [15].

Study data from each patient were prospectively documented in a case report form (CRF). CRFs were documented pseudonymously and included a patient identification code, date of birth, sex, date of admission, date of discharge, diagnosis, symptoms, devices, location of swabs taken on admission, pathogens detected, location of detected pathogens, medication, antibiotic prophylaxis, immunosuppressive therapy and occurrence of any NIs. NIs were defined as infections occurring ≥48 hours after admission and refer to any systemic or localised conditions that result from the reaction to an infectious agent or toxin [12]. If NIs were detected, a special form was completed containing information concerning the type of NI, detected pathogens, and treatment. In addition, the unit nurses completed a midnight census every day to obtain information about the number of inpatient days and utilisation days for devices (tracheostomy, feeding tube/PEG, urinary tract catheter, and central-venous catheter), parenteral nutrition, and mechanical ventilation. 

### Ethics

The screening of all patients of the PPC unit before admission was part of the evaluation of a standard procedure of the PPC unit according to a prospective quality audit. Surveillance data and patient information were extracted and recorded in a case report form, and were then entered into an anonymous SPSS data sheet. Therefore, informed consent of patients and parents was not required. No ethical approval was requested [16], [17], [18]. This procedure is justifiable because the smears are part of the standard procedure in the unit. 

### Statistical analysis

Descriptive statistics were used to analyse the characteristics of study participants, pathogens upon admission, location of pathogens, proportion of carrier of MDR pathogens upon admission, and occurrence of NI. NI incidence density was normalized to 1,000 inpatient treatment-days as follows: 

NI incidence density=no. of NIs/patient days x 1000

Statistics were performed using IBM SPSS^®^ Version 23.

All analyses except the NI incidence density were analysed on the patient level (n=118). For the analysis of NI incidence density, the data of all admissions were included (n=198).

## Results

During the study period, there were 198 admissions to the PPC unit and 118 different patients were admitted. 38.8% (n=75) of the 118 patients were admitted more than once (20% twice, 9% three times, 5% four times, 3% five times and 1% six times). Demographic characteristics of the 118 different patients are presented in Table 1 (Tab. 1).

### Pathogens and MDR pathogens

In 74 (63%) of 118 patients, the screening yielded at least one pathogen (Table 2). A total of n=21 patients were colonised with an MDR pathogen upon admission (18% of all patients and 28% of those who are colonised with at least one pathogen). 

Five children (24% of those with MDR pathogen colonisation, 7% of those carrying a pathogen and 4% of all patients) were colonised with more than one MDR pathogen upon admission. The most commonly detected MDR pathogens were *E. coli* (8%) and Pseudomonas spp. (8%) (Table 2 (Tab. 2)).

### Location of pathogens

**Figure 1:**
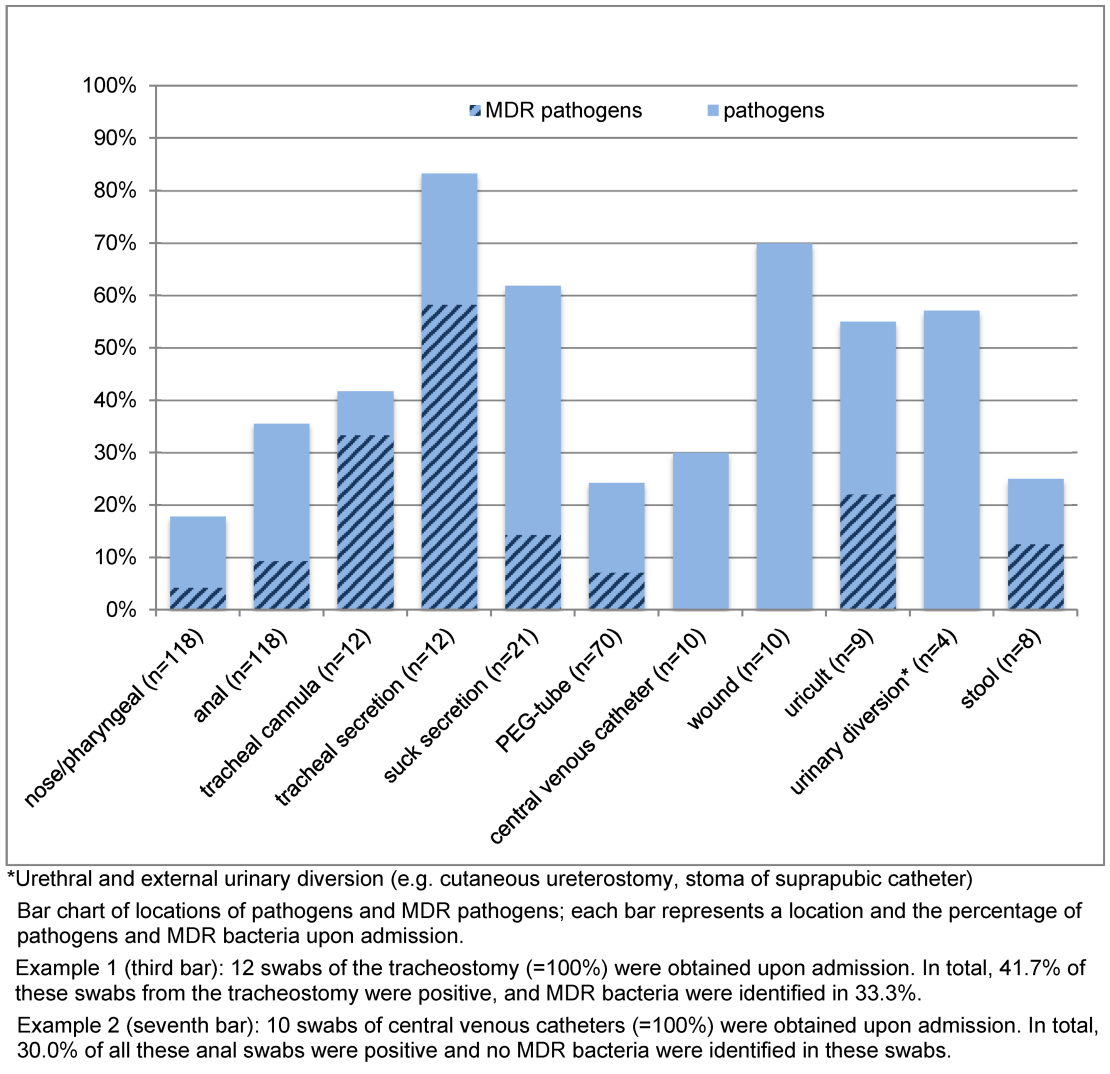
Location of pathogens

Patients wearing a tracheal cannula exhibited the highest risk of colonisation with pathogens. In total, 83% (n=10) of all 12 patients with a tracheal cannula harboured a pathogen in their tracheal secretions (Figure 1 (Fig. 1)).

### Location of MDR pathogens

In 33% (n=4) of patients with a tracheal cannula, the swab of the tracheal cannula yielded an MDR pathogen; 58% (n=7) had an MDR pathogen in their tracheal secretions (Figure 1). Only 7% (n=5) of the 70 patients with a PEG tube had an MDR pathogen located at the PEG exit site, and none of the swabs taken upon admission from central venous catheter exit sites (n=10) yielded MDR pathogens.

### Nosocomial Infections during the study period

The NI incidence density was analysed for all cases admitted to the unit during the one-year surveillance (n=198). Two patients (1%) had a total of two documented NIs. One patient had a respiratory tract infection, and one had a skin/soft tissue infection; both were due to methicillin-sensitive *S. aureus*. No NIs due to MDR pathogens were observed during the 12-month observation period. The NI incidence density was 0.99/1000 inpatient treatment days.

## Discussion

To our knowledge, this is the first surveillance investigating the prevalence of MDR pathogen colonisation and NI in a German PPC unit. The most important findings derived from this study were a high prevalence of MDR pathogen colonisation upon admission to the PPC unit (18%), a 58% prevalence of MDR pathogen colonisation in tracheal aspirates of patients with tracheostomy, and a low NI incidence density (0.99 events/1000 inpatient treatment days) with no NIs caused by MDR pathogens.

The basic characteristics of advanced PPC patients exhibit some similarities to those of paediatric intensive care unit (PICU) patients in terms of high utilisation rates of devices and intensive exposure to antimicrobial treatment (in the medical history of PPC patients) [7], [8]. In addition, some of the PPC patients are immunocompromised due to oncological or autoimmune disease and their respective treatment. Hitherto, the prevalence of MDR pathogen colonisation has not been thoroughly investigated in German PICUs. Published data on the prevalence in PICUs from other countries must be interpreted with caution, given that the epidemiology of MDR pathogen colonisation as well as the incidence of MDR pathogen NI differs substantially between different countries. Dedic-Ljubovic and Hukic [19] as well as Folgori et al. [20] reported that 44% of children were colonised with MDR bacteria upon admission to a PICU. Jaworski et al. (21), who investigated the colonisation of MDR pathogens in paediatric cardiac patients, reported an admission prevalence of 9%. The most common MDR pathogens in their population were ESBL-producing *Enterobacteriacea*e-like *Klebsiella pneumoniae* (3.9%) and *E. coli* ESBL (3.2%) [21]. 

Concerning the prevalence of MRSA colonisation upon admission (4.1% in our patient cohort), studies performed in palliative inpatient care facilities for adults from Germany [22], [23], the republic of Ireland [24] and Saudi Arabia [25] reported results up to 12%. There is only one study (Heckel et al. [23]) providing prevalence data on MRGN in (adult) palliative care. Those authors reported a 4.1% prevalence of MRGN in adult palliative care patients. However, that study focussed on MRSA, and only provided MRGN prevalence as an incidental finding; it was limited by the fact that systematic screening for MRGN was not performed. Therefore, a comparison with the higher MRGN prevalence found in our study is not possible. 

Remarkably, in our sample, patients with PEG tubes did not have high rates of MDR pathogens upon admission. Thus, the presence of a PEG tube will not be rated as a risk-factor for colonisation with MDR pathogens in our future patients. Contrary to the PICU studies published by Almuneef et al. [26] and Elward and Fraser [27], patients with a central venous catheter did not have high rates of MDR pathogen colonisation or NIs in our patient cohort. However, our rate of patients with a long-term central venous catheter was very low. The rate of colonisation with MDR pathogens in patients with tracheostomy was 58% in tracheal secretions. Our results indicate that (from the perspective of barrier precautions to avoid nosocomial transmission) special attention must be paid to patients with tracheostomy. Screening for MDR pathogens in patients with tracheostomy admitted to a PPC unit may therefore be of great importance to avoid NIs and nosocomial transmissions. Strict single-room barrier nursing should be performed until screening results are available. 

Concerning the prevalence of MDR pathogens upon admission (18% in our patient cohort), studies performed in paediatric long-term care facilities reported increased results [28], [29]. Similarly, the overall incidence of NIs in paediatric long-term care facilities is much higher [30]. 

The overall incidence of NIs was 0.99/1,000 inpatient treatment days. Compared with data on NIs in German neonatal intensive care units (NICU), this rate is very low (6/1000 patient days) [31]. No NIs due to MDR pathogens and no transmission events of MDR pathogens to different patients were observed. This finding may reflect that the implementation of our hygiene approach has helped to contain nosocomial transmission of pathogens between patients. To provide definitive evidence, further prospective studies with a different research design are needed. 

An important prerequisite of our concept is that all members of the PPC treatment team (from the chief physician to housekeeping personnel and volunteers) are thoroughly educated and trained not only in standard hygiene precautions but also in the implementation of additional adjusted barrier precautions. Patients and/or their caregivers/parents are informed about this concept upon admission; the informative hand-outs are available in different languages to overcome language restrictions. Little is known about the training provided to staff in adult units. However, two surveys regarding the management of MRSA in adult palliative care units and hospices reported that staff training was provided in only 59% [32]. In 84% of the units that responded to the survey, patients who are colonised with MRSA received information about MRSA, the information given was typically verbal [33] and more likely to be imparted if a patient was infected rather than colonised [32]. 

Next to hand hygiene and the use of contact precautions until patients are culture-negative [34], the training of staff members, patients, and families seem to be an important measure to prevent NIs due to MDR pathogens [35], [36]. The relevance of single-room isolation is still a matter of debate [34], [37], and some studies noted that strict single-room isolation may expose the patient to an increased risk of medical complications [38], [39] as well as anxiety reactions and depression due to contact precautions [39], [40]. Particularly in the field of adult palliative care, the impact of contact precautions on patients and family caregivers’ quality of life is discussed in the literature. Datta and Juthani-Mehta [41] actually consider the removal of contact precautions from palliative care settings altogether. However, contrary to adults in palliative care units, who often suffer from terminal conditions and are bedridden, children receiving treatment in a PPC unit have an average life expectancy of several years. Therefore, nosocomial transmissions need to be prevented. To enhance social participation for patients colonised with MDR pathogens during their time in the unit and simultaneously prevent nosocomial transmissions of MDR pathogens, adjusted hygiene concepts that enable the affected patients/families to participate should be applied in PPC units.

Even if there are no studies to our knowledge regarding the following approaches, further risk factors especially for the patients on the PPC unit may derive from dog therapy and intensive direct contact with visitors (e.g., siblings, grandparents) and volunteers. More research is necessary in these fields to defend against transmission of MDR pathogens. 

Our study had some limitations. First, screening was not performed on the day of discharge. Therefore, the issue of nosocomial transmission of MDR pathogens could not be addressed sufficiently. Further, some of the screenings were done immediately prior to admission (by the general practitioner/outpatient paediatrician) and some on the day of admission. Moreover, this was a small study which did not have the power to identify risk factors for colonisation with MDR pathogens. Finally, the study was performed in a single PPC institution, and the results may not be generalisable to other PPC units.

## Conclusions

In conclusion, due to high prevalence, it is reasonable to screen PPC patients for MDR pathogen colonisation upon admission. Special attention must be given to patients with tracheostomy. To enhance social participation for patients colonised with MDR pathogens during their time on a PPC unit, adjusted hygiene concepts that enable the affected patients/families to participate have to be established. Further research including MDR screening on the day of discharge is necessary to validate the adjusted hygiene concept.

## Notes

### Competing interests

The authors declare that they have no competing interests.

### List of abbriviations

NI – nosocomial infection

PPC – paediatric palliative care

LLC – life-limiting conditions

MDR – multidrug-resistant

MRSA – methicillin-resistant Staphylococcus aureus

MRGN – multidrug-resistant Gram-negative pathogens

CRF – case report form

PEG – percutaneous endoscopic gastrostomy

ACT – Association for Children with Life-threatening or Terminal Conditions and their Families

NICU – Neonatal intensive care unit

### Author Contributions 

CH, AS, CG and BZ conceived and designed the experiments; 

PS and CH performed the experiments; 

PS and CH analysed the data; 

AS, CG, JW and BZ supported the interpretation of data

PS, CH, AS, CG, JW and BZ wrote the paper

## Figures and Tables

**Table 1:**
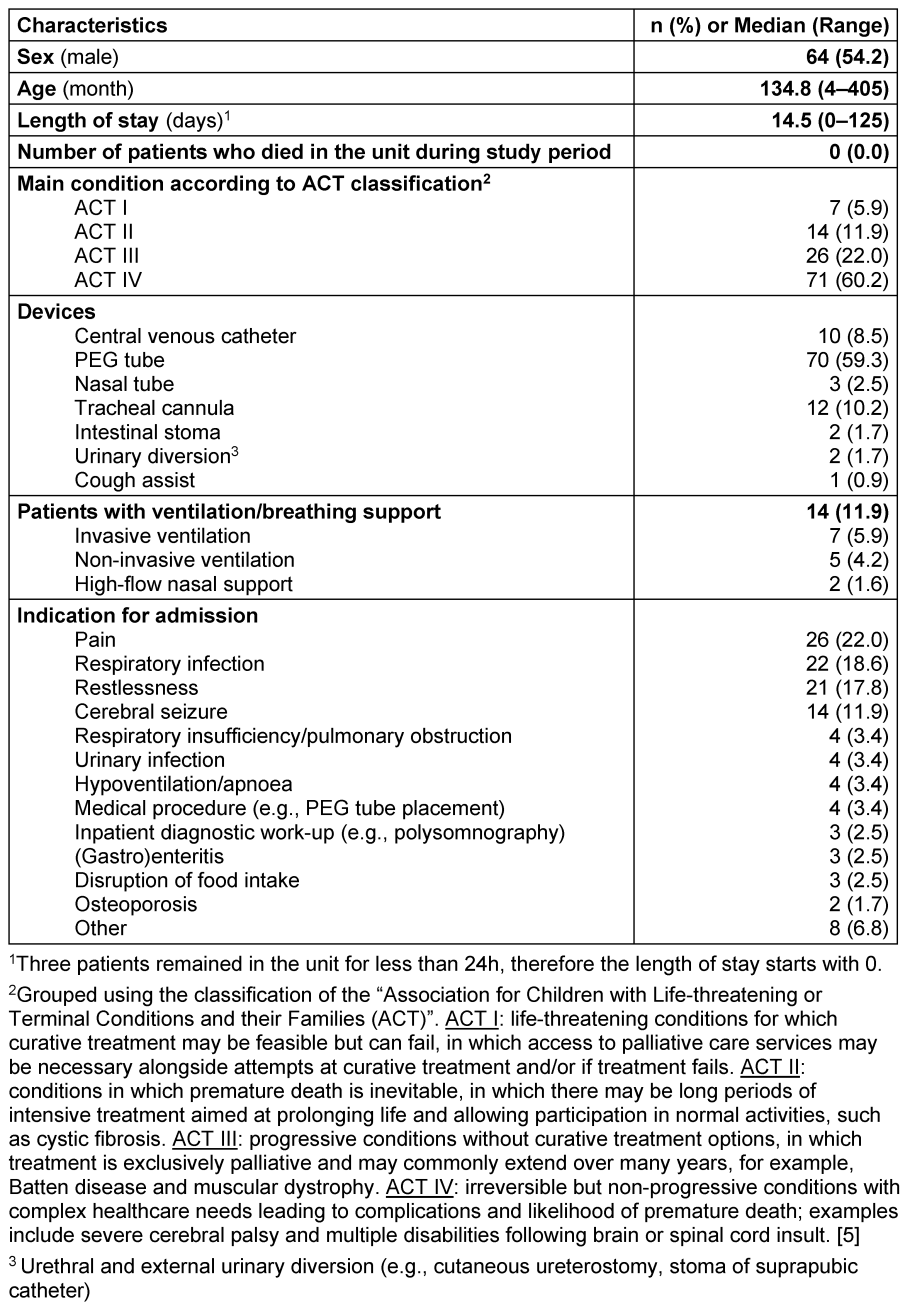
Patient demographics (n=118)

**Table 2:**
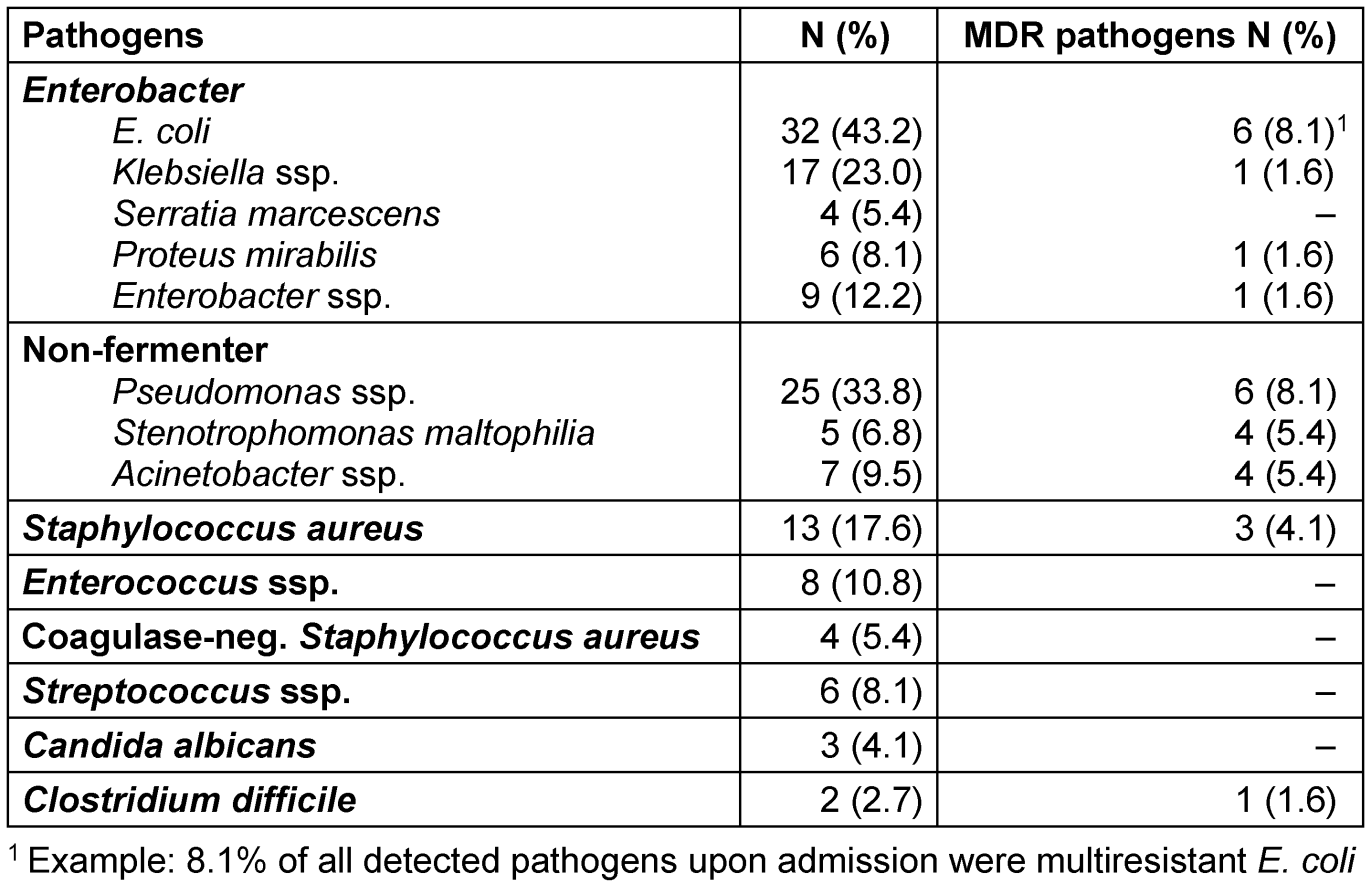
Pathogens and MDR pathogens upon admission (n=74 colonised patients)
